# Selectivity of stimulus induced responses in cultured hippocampal networks on microelectrode arrays

**DOI:** 10.1007/s11571-016-9380-6

**Published:** 2016-02-22

**Authors:** Alexey Pimashkin, Arseniy Gladkov, Ekaterina Agrba, Irina Mukhina, Victor Kazantsev

**Affiliations:** Neuroengineering Laboratory, Translational Technologies Center, Lobachevsky State University of Nizhny Novgorod, Gagarin ave 23, Nizhny Novgorod, Russian Federation 603950; Central Research Laboratory, Cell Technology Department, Nizhny Novgorod State Medical Academy, 10/1 Minin and Pozharsky Square, Nizhny Novgorod, Russian Federation 603005

**Keywords:** Neural networks, Microelectrode array, Electrical stimulation in vitro, Hippocampal cultures, Brain information decoding

## Abstract

Sensory information can be encoded using the average firing rate and spike occurrence times in neuronal network responses to external stimuli. Decoding or retrieving stimulus characteristics from the response pattern generally implies that the corresponding neural network has a selective response to various input signals. The role of various spiking activity characteristics (e.g., spike rate and precise spike timing) for basic information processing was widely investigated on the level of neural populations but gave inconsistent evidence for particular mechanisms. Multisite electrophysiology of cultured neural networks grown on microelectrode arrays is a recently developed tool and currently an active research area. In this study, we analyzed the stimulus responses represented by network-wide bursts evoked from various spatial locations (electrodes). We found that the response characteristics, such as the burst initiation time and the spike rate, can be used to retrieve information about the stimulus location. The best selectivity in the response spiking pattern could be found for a small subpopulation of neurones (electrodes) at relatively short post-stimulus intervals. Such intervals were unique for each culture due to the non-uniform organization of the functional connectivity in the network during spontaneous development.

**Electronic supplementary material:**

The online version of this article (doi:10.1007/s11571-016-9380-6) contains supplementary material, which is available to authorized users.

## Introduction

Selectivity is one of the key properties of brain dynamics necessary for the classification of sensory information. It has been shown that selectivity requires two basic properties of a sensory stimulus response, namely high temporal precision of spike times (Jenmalm and Johansson 1997; Wesson et al. 2008) and firing rate (Adrian and Zotterman 1926; Celebrini et al. 1993). A neuron’s selectivity to different stimuli has been well studied in the visual cortex (Sigala and Logothetis 2002; Maunsell and Van Essen 1983; Crook et al. 1998; Sompolinsky and Shapley 1997), intraparietal area (Fanini and Assad 2009), somatosensory cortex (Wilent and Contreras 2005) and temporal cortex (Kraskov et al. 2007). Most of these works have been done using single cell or single unit (electrode) recording techniques. However, selective responses at the level of single cells need to be linked to the dynamics of a population of interconnected neurons. Several studies of decoding visual stimuli using multisite recordings in vivo revealed important spatio-frequency features of local field potentials (Seif and Daliri 2015; Wang et al. 2008) or spiking patterns (Sundberg et al. 2009). Visual stimulus encoding in neural networks was also studied in retinal ganglion cells placed on microelectrode arrays (Gong et al. 2010). It has been established that unique features of the sensory stimuli in the brain can be found in time–frequency domain, in particular, a precise spike timing of the initial activity of the stimulus responses, firing rate and particular frequencies of the responses (gamma, theta) etc. Note that in these studies (in vivo and in vitro) the stimuli were uniformly applied to the whole neural network (light or visual image). However it was shown that information processing implemented in dendrites and synapses (Yoneyama et al. 2011; Engel et al. 2005; Rasch et al. 2009). Thus the spatial features of the information encoding/decoding in the neural networks can be studied applying localized stimulus to the neurons or small subpopulations (electrodes).

Many experimental observations of neural network activity have reported the existence of repeatable spiking patterns both in vivo (Ikegaya et al. 2004; Mokeichev et al. 2007) and in vitro (Tateno and Jimbo 1999; Raichman and Ben-Jacob 2008; Segev et al. 2002; Madhavan et al. 2007; Pimashkin et al. 2011). These spiking patterns consisted of motifs organized by the coherent activation of many neurons. Such repeatable patterns could be treated as a tool to encode information in the characteristics of spike sequences. In this context, the configuration of these patterns should be sensitive and selective to the external stimulation.

In network studies, there is an expanding interest in in vitro models of dissociated neuronal cultures grown on microelectrode arrays (MEA). MEA systems allow for the simultaneous recording and stimulation of electrophysiological activity at multiple sites non-invasively (Martinoia et al. 2005; Jimbo et al. 2000; Bove et al. 1995; Jimbo 1992). During development, dissociated cultures form networks of synaptically coupled neurons capable of generating electrical activity that can be recorded by multiple extracellular electrodes. Changes in the network architecture can be simultaneously monitored by optical microscopy. Thus, culture networks represent a convenient experimental model to analyze network mechanisms of neuronal response selectivity. In mature cultures, low-frequency (0.05–0.3 Hz) biphasic pulse stimulation of single or paired electrodes can induce burst responses within several hundreds of milliseconds of the stimulation (Bakkum et al. 2008; Maeda et al. 1995; Wagenaar et al. 2005). These stimulus-evoked bursts have further been used for the analysis of spike timing precision (Shahaf et al. 2008; Potter et al. 2005) and learning and memory (Shahaf and Marom 2001; Le Feber et al. 2010). In recent studies, it has been shown that low-frequency electrical stimulation could induce changes in the inter-burst interval (Bologna et al. 2010) and modify the spiking structure of spontaneously generated bursts (Vajda et al. 2008). Other studies, however, have reported that continuous low-frequency stimulation did not significantly affect spike sequences within the response (Chiappalone et al. 2008; Eytan et al. 2003). However, strong tetanic stimulation delivered through two distant electrodes with specific delays could significantly change the structure of the burst response (Wagenaar et al. 2006; Chiappalone et al. 2008; Tateno and Jimbo 1999). Interestingly, the stimulus selectivity relative to the stimulation site has been reported in the population response over electrodes for the burst initiation time profile (Shahaf et al. 2008).

In a recent study using novel setup with high-density microelectrode arrays (4096 with 21 µm electrode size) it was shown that bursting activity propagate through short and preferably locally distributed pathways (Maccione et al. 2012). Such method allowed to monitor spiking activity with a single cell precision. During network-wide bursts formation the spikes propagation recruited mostly sequence of small clusters of nearby cells (tens of cells) within a each small time interval of several milliseconds. Furthermore we suggested that the selectivity implemented on the scale of small neuronal subpopulations (tens of microns) and can be investigated with conventional 60 electrodes arrays with 50 µm electrode size.

In this study, we demonstrate that selectivity features of the response can be used to retrieve the stimulus location on the basis of the precise spike latency and the firing rate of the stimulus evoked response. We further analyzed how efficient the selectivity is depending on the size of the neural network (i.e., provide population coding). We characterized the stimulus response as the activity at individual electrodes over short time intervals (20 ms), which displayed selectivity properties in different time intervals and spatial localizations of the response.

## Materials and methods

### Cell culture

Hippocampal cells were dissociated from embryonic mice (E18) and plated on microelectrode arrays (MEAs) pre-treated with the adhesion promoting molecule polyethyleneimine (Sigma P3143) at a final density of approximately 15,000–20,000 cells/mm^2^. C57Bl/6 mice were euthanized via cervical dislocation, according to the protocols approved by the Russian National Ministry of Public Health for the care and use of laboratory animals. The embryos were removed and decapitated. The entire hippocampi, excluding the cortex, whole medulla and the lower part of the pons, were dissected under sterile conditions in Ca^2+^- and Mg^2+^- free phosphate-buffered saline (PBS-minus). Following enzymatic digestion for 20 min with 0.25 % trypsin at 35.5 °C (Invitrogen 25200-056), the cells were separated by trituration (50 passes) using a 1 mm diameter of pipette tip. The solution was then centrifuged at 1000 rpm for 4 min and the cell pellet was immediately re-suspended in Neurobasal medium (Invitrogen 21103-049) supplemented with B-27 (Invitrogen 17504-044), glutamine (Invitrogen 25030-024) and 10 % fetal calf serum (PanEco К055). The dissociated cells were seeded in a 25–30 μl droplet covering the center of the culture dish within the 1 mm^2^ electrode region of the MEA, forming a dense monolayer (Pimashkin et al. 2013). After the cells had adhered (usually within 1.5 h), the dishes were filled with 1 ml of Neurobasal medium (NBM) supplemented with B-27 and 0.5 mM glutamine with 5 % fetal calf serum. After 24 h, the plating media was replaced with NBM containing 0.5 mM glutamine and 0.4 % fetal calf serum, but with no antibiotics or antimycotics. Glial growth was not suppressed, because glial cells are essential for the long-term health of the culture. One half of the media was changed every 2 days. The cells were cultured under constant conditions of 35.5 °C, 5 % CO_2_ and 95 % air at a saturating humidity in a cell culture incubator (MCO-18AIC, SANYO).

Phase contrast images of the cultures were taken weekly to record the status of the culture using a Leica DMIL HC (Germany) inverted microscope with a 10×/0.2Ph1 objective. Experiments were performed when the cultures were 3–5 weeks in vitro.

### Electrophysiological methods

**Fig. 1:**
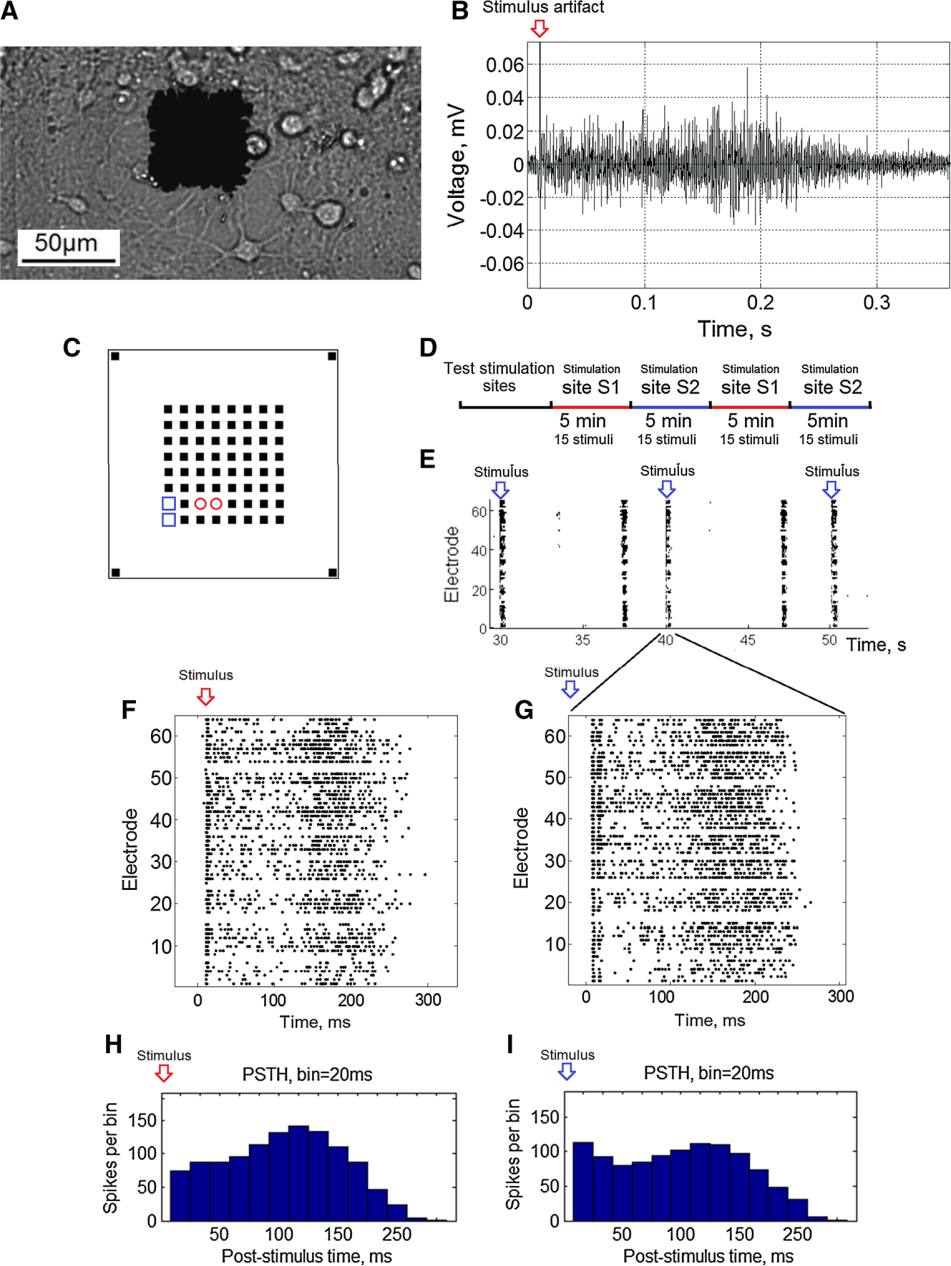
**a** Hippocampal neurons cultured on a MEA with 64 electrodes of 50 μm size. **b** Typical electrophysiological signals recorded from a single microelectrode during a stimulus response. **c** Location of the stimulation sites (pairs of electrodes), S1 and S2, on the MEA. **d** The stimulation protocol; each site was stimulated twice for 5 min (see “Materials and methods” section). **e** Raster plot of the sample activity (20 s) with spontaneous and stimulus evoked bursts recorded over 64 electrodes. Each *black* point on the raster represents spike. *Raster plots* of stimulus response examples evoked from sites S1 (**f**) and S2 (**g**). Post Stimulus Time Histograms (PSTH) with 20 ms time bin of the response activity within 300 ms of stimulation at sites S1 (**h**) and S2 (**i**), respectively

Extracellular potentials were collected using 64 planar platinum black electrodes integrated into the MED64 system (Alpha MED Science, Japan). The microelectrode arrays (MEA) had 8 × 8 (64) electrodes with a size of 50 µm × 50 µm and were spaced by 150 µm (Fig. 1a). Data were recorded simultaneously in 64 channels at a sampling rate of 20 kHz/channel. Electrical stimulation was applied using a STG-4004 stimulator (Multichannel Systems, Germany). All signal analysis and statistics were performed using custom made software in Matlab^®^.

### Spike detection

The detection of recorded spikes (Fig. 1b) was based on a threshold calculation:

1 $$T = N_{S} \sigma ,$$

where $\sigma = median\left( {\frac{|x|}{0.6745}} \right)$ which was the estimate of the median normalized to standard deviation of a signal with no spikes (see Quiroga et al. 2004 for more details), *x* is the band pass-filtered (0.3–8 kHz) signal and $N_{S}$ is the spike detection coefficient which was set to 8. The amplitudes of detected spikes were in the range of 10–40 μV.

We applied spike-sorting algorithms to classify spikes coming from different cells (Quiroga et al. 2004). We found that during high-frequency bursting discharges integrated signals from a large area with group of the neurons (50 μm electrode size) couldn’t be differentiated as local spikes arriving with negligible latencies (Support Fig. 1, cluster 3). Only spikes between the bursts had distinguishable shapes (Support Fig. 1, cluster 1). Thus, we analyzed the signals contributed by a local group of the neurons near a particular electrode as a single event.

### Stimulation protocol

**Fig. 2:**
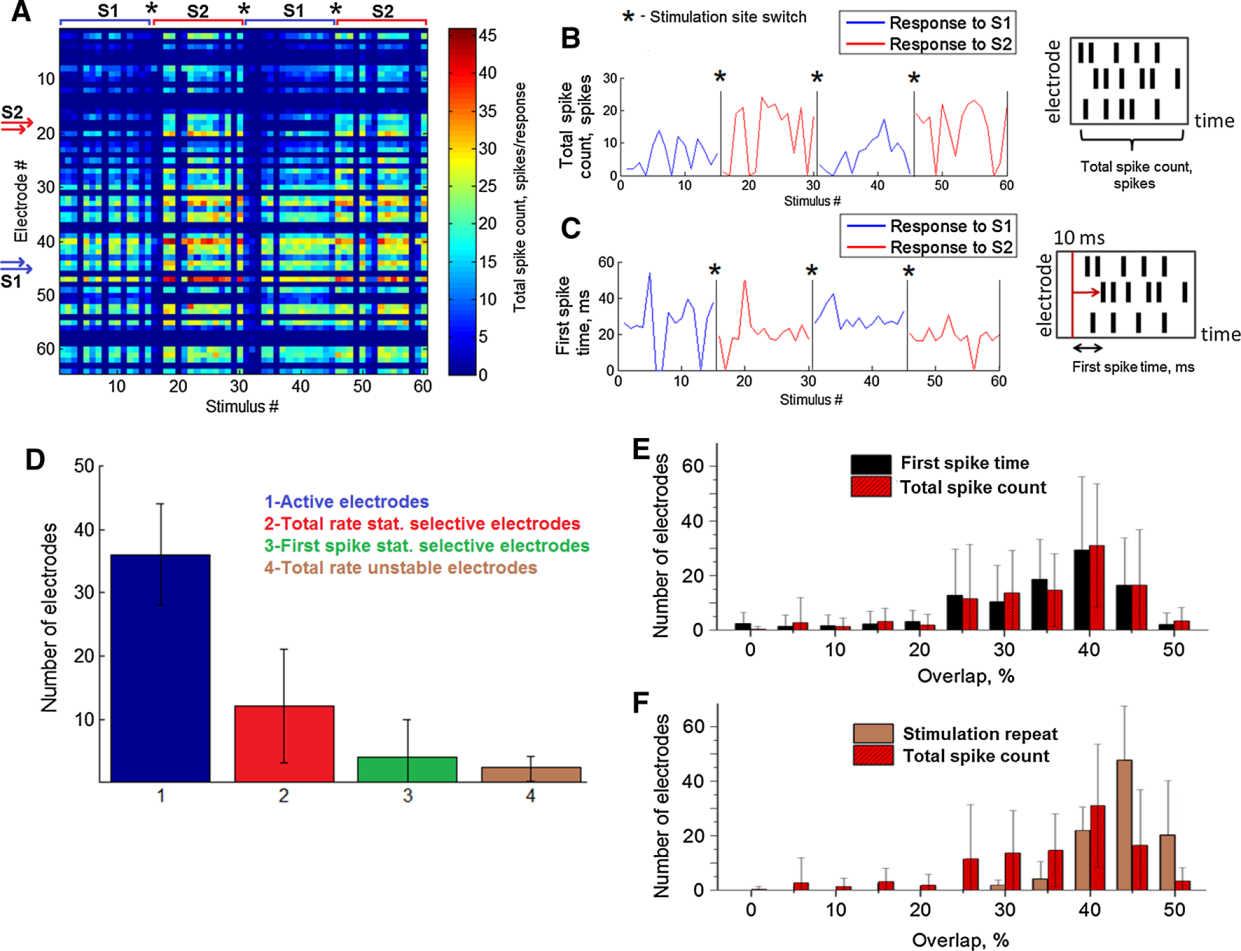
**a** Time course of the responses to the stimuli of two different sites (S1 electrode pairs 18–19 and S2 50–51) during trial stimulation. Each site was stimulated twice by 15 stimulus pulses with 800 mV amplitude. *Color* represented total spike count for each electrode in each response (see “Materials and methods” section). Example time courses for the **b** total spike count measure and **c** burst activation times observed in a single electrode #17 after each 300 ms post-stimulus interval during repetitive stimulation of sites S1 and S2. *Asterisk* (*) depicts time where stimulation site was switched to another. **d** The number of active electrodes, statistically selective electrodes identified using the spike rate measure (*2*) and burst activation times (*3*) and number of electrodes with unstable responses showing different responses after second stimulation of the same sites (see “Materials and methods” section). **e** Distributions of the overlaps calculated from the spike rate measure of the responses from two different sites (*red bars*) and responses from repetitive stimulation of the same sites (*brown bars*). **f** Distributions of the overlaps calculated from the spike rate measure and burst activation times. Stimulation repeat—overlaps of the responses evoked from single sites which were stimulated two times with 5 min each. Total spike count—overlaps of the responses evoked from different sites, same as presented on **e**. (Color figure online)

Electrophysiological activity was induced using a train of biphasic voltage pulses of 400–800 mV with a 600 μs duration. The stimulus was delivered through a pair of nearby electrodes, for which the pulse of one electrode was antiphasic to the other to localize the induced current flowing between the electrodes. Such electrode pairs were defined as the stimulation site. All experiments were performed in the presence of spontaneous bursting activity. The observed inter-burst interval was in the range of 10–20 s. The inter-stimulus interval for each culture was set to match the mean inter-burst interval. This method maximized the probability of evoking a burst response. Two stimulation sites (S1 and S2) were chosen so that the inter-electrode distance was 2–4 electrodes (400–800 μm). For each experiment, the stimulation site was chosen according to its ability to induce population bursts in response to more than 80 % of the stimuli. Among 20 tested sites for each MEA, only 4 sites on average evoked stable responses. A stimulation trial consisted of the consequent stimulation of each site two times for 15 stimuli (5 min) (Fig. 1d). 30 stimuli (N_a_ or N_b_) at each site were applied in total. Such repetitive stimulation was used to investigate the selectivity as a stable property rather than an alteration in the synaptic connectivity when switching between the sites (Fig. 2a)

### Evoked response analysis

The stimulation of each site induced a population response in the form of a burst of activity over most of the electrodes. Raster plots of the responses of two distinct sites for an example culture are shown in Fig. 1e–g. To characterize the evoked bursts we used a Population Post-Stimulus Time Histo-gram [PSTH, (Chiappalone et al. 2008; Cozzi et al. 2006; Jimbo et al. 2000; Li et al. 2007; Marom and Shahaf 2002; Shahaf et al. 2008; Stegenga et al. 2010; Wagenaar et al. 2004)] (Fig. 1h, i). Within each 20 ms time bin of the post-stimulus response, we calculated the total number of spikes recorded from all of the electrodes.

Selectivity was defined as the ability of neurons to generate *unique* responses to *different* stimulus locations (i.e., stimulation sites). We investigated two main features of the responses, including the *activationtime,* which represents the latency of the first synaptically evoked spike, and the *spike rate,* representing the number of spikes in the post-stimulus spiking activity (300 ms). Note that the spikes within the first 15 ms after stimulus have non-synaptic origin and only latter activity was considered as synaptically evoked spikes (Bakkum et al. 2008; Jimbo et al. 2000; Marom and Shahaf 2002; Wagenaar et al. 2004). The latter interval was binned into 20 ms time slots (bins). The number of spikes in each bin per electrode was defined as the *spike rate pattern*. Different patterns relative to different stimulation sites were collected and analyzed. Note that we considered only those time bins where the evoked responses were stable, (i.e., reproducible). Further analysis was performed for the time bins in which at least 80 % of the stimuli evoked at least one spike. There was also a condition for responding electrodes, i.e., for electrodes eliciting at least one spike within 300 ms after the stimulus in more than 80 % of the cases. The presence of *statistical selectivity* for each electrode was defined if two sets of the responses from the electrode were statistically different applying Mann–Whitney rank sum test (*p* < 0.001).

We also quantified the selectivity for each electrode as a level of discrepancy between two sets of the responses to different sites using clustering methods. We applied K-Means clustering to all responses in the trial stimulation which optimally separated the sequence of the responses into two data sets. Then the set of the responses with known stimulus sources (sets A and B) we compared with set of the responses after cluster analysis (A′ and B′). Number of the patterns incorrectly identified according to its stimulus source (N_a_ = A′ ∩ B and N_b_ = B′ ∩ A) represents a classification error. A percent of such patterns relative to a total number of the responses [100 · N_c_/(N_a_ + N_b_)] was defined as an overlap for the selectivity measure and varied in range 0–50 %. The overlap was calculated for each pair of sets of the electrode responses and for each time bin from each electrode. In contrast to statistical test such measure represented a degree of the selectivity, e.g. 0 % overlap can be found if all values of the responses from one stimulation site are greater than the responses from other stimulation site or vice versa. Overlap equal to 10 % will be found if 10 % stimuli applied to both sites evoke undistinguishable responses.

### Pattern classification

To estimate the selectivity of the responses from all electrodes, we applied the following procedures.

First, the patterns of the total spike count from each electrode were united in a single set of patterns that was further clustered into two groups using a *K*-*means clustering method*. Then, the initial set of response patterns to the different stimulations was compared with the clustered set to validate the classification method and estimate the classification accuracy. The fraction of patterns correctly identified was considered as the K-means classification accuracy. The accuracy was also estimated for other response characteristics, including the spike rate pattern and burst activation time. A conventional K-means clustering method separates patterns into clusters according to the minimum Euclidean distance to the cluster centroid. The cluster centroid was found using efficient heuristic algorithms.

Next, we applied a modified K-means clustering method where each cluster centroid was preliminary calculated by averaging the responses for each electrode. We defined this procedure as a K-means clustering with predefined centroids (*K*-*means p.c.*). The cluster centroids were estimated by averaging the values of each response from each electrode for a subset of the particular stimulation source-induced patterns. Then, each pattern was assigned to one of the two clusters according to the minimum Euclidean distance to one of the cluster centroids.

Finally, the clustering accuracy was tested using the *Support Vector Clustering* (SVC) method with a Gaussian Radial Based Function kernel. The kernel parameter and confidence intervals were set using a fivefold cross-validation procedure. First, the method was trained to classify different patterns into two data sets. Next, we examined the classification accuracy by cross-validation.

## Results

We first analyzed how electrical stimuli (a train of 10–20 s inter-stimulus interval) applied to different stimulation sites (electrodes pairs, Fig. 1c) could evoke statistically distinguishable response spiking patterns on a microelectrode array (Fig. 1f, g). The response spiking activity was evoked by consecutive stimulation trains of two stimulation sites (Fig. 1d). Stimulation sites S1 and S2 were chosen according to their ability to generate population burst responses. Such response typically consisted of a short period (100–300 ms) of spiking activity from recorded from the electrodes (Fig. 1b). Raster plots and post-stimulus histograms (PSTHs) of the responses from two stimulation sites are illustrated in Fig. 1f–i, respectively. Shapes of the PSTHs represented average time course of network-wide activity in each 20 ms time bin. Such dynamics was also typical for spontaneous activity.

### Selectivity at individual electrodes

Figure 2a depicts time course of the total spiking count of the responses from all electrodes during trial stimulation of two sites. Color of each response represented the total number of spikes after stimulus. On average, the number of *activeelectrodes* was 36.18 ± 8.44 SD out of 64 electrodes in total and estimated from 50 stimulation trials i.e. recordings. We tested stationarity of the responses during repetitive stimulation of each site according to the stimulation protocol. Two sets of the responses as total spiking count evoked from single sites were tested for statistically significant difference. Note that we took into account the responses that has at least one spike in the evoked bursting activity. For example in Fig. 2b sets of the responses were tested from S1 (responses 1–15 and 31–45) and S2 (responses 16–30 and 46–60) independently. Only the electrodes with stationary responses evoked by each stimulation site were used in further analysis. In summary we found that 4.1 ± 1.12 (2.1 % ± 1.9 SD %) of all active electrodes were not stable during trial stimulation. Various cultures show different ability of the response phase-locking to the stimuli (Fig. 2b) due to high variability of spontaneous bursting intervals and hence the spiking rate of the bursting responses was not stable in stimulation series on some cultures.

Next, we analyzed the selectivity with respect to a single electrode’s response activity. We were interested in whether the neurons could respond to different stimuli with separable and repeatable features of the response signals. The responses for each electrode can be characterized by two main features, including the burst activation time, i.e., the latency to the first synaptically evoked spike following a stimulus, and the total spiking count of the response within the first 300 ms post-stimulus period.

An example of the total spike counts from one electrode during a stimulation trial of sites S1 and S2 is shown in Fig. 2b. If the values from two sets of the spike rates were significantly different, then the electrode (i.e., neurons contributing to the electrode signal) was considered to be *statistically selective* (see “Materials and methods”) to the stimulation sites using the total spike count measure. Note that the statistical selectivity test indicated only the difference in the median values of the observed data sets. To estimate the degree of the selectivity we calculated an *overlap* characteristic for each electrode (see “Materials and methods” section). Such a characteristic represents ambiguity in the responses classification of the two response sets. The *overlap* between the spike rates from the example shown in Fig. 2b was 18 %, indicating that the stimulation source couldn’t be correctly identified in 18 % of the data.

Next, we analyzed the selectivity with respect to the burst activation time. Figure 2c depicts the time of the first post-stimulus spikes during the stimulation of sites S1 (Fig. 2c, blue line) and S2 (Fig. 2c, red line). In this example, the sets of spike times were significantly different; electrodes with such responses were considered as *statistically selective* with respect to their burst activation time. The sets of spike times were also characterized by their overlap. For the above example, this overlap was 23 %.

The summarized results of the statistical selectivity tests for single electrodes are shown in Fig. 2d. The experiments were performed on 11 cultures where each culture was stimulated from age 20–35 days in vitro (DIV). During this period, different stimulation sites were chosen for trial stimulations so that each culture was stimulated with 4–5 trials (50 trials in total). Note that the spike rate and burst-evoking efficacy for various electrodes changed on a timescale of 5 days due to the spontaneous development of the culture. The number of active electrodes varied in all cultures and in average was 36.12 ± 7.78 (SD) out of 64 electrodes of the MEA. We found that the number of *statistically selective* electrodes with respect to the burst activation times was relatively small, 3.89 ± 5.31 % SD (10.77 % of all active electrodes). Surprisingly, the average number of *statistically selective* electrodes with respect to the spike rate measure was quite high, 11.89 ± 6.89 SD, which was 32.93 % of all active electrodes. These results indicate the efficiency of neurons to generate responses with distinguishable spike rates. The difference between the mean characteristics was not statistically significant (*t* test) which can be explained by high variability between cultures.

The distribution of the overlaps with respect to the total spike counts measure and burst activation time for all experiments (11 cultures and 50 trials) are presented in Fig. 2e. Interestingly, the distributions for both response measures were statistically similar, and most of the electrodes had overlaps in the range of 25–45 %. Note that the number of electrodes with an overlap of less than 5 % was close to 0 (for the activation time: overlap <5 %, 2.42 ± 4.02 SD; for the total spike count: overlap <5 %, 0.34 ± 1.04 SD). These results indicate the absence of error-free selectivity in single electrodes. During trial stimulation each stimulus response may be affected by spontaneous bursting activity.

We also tested the stability of the responses evoked from single sites during stimulation (Fig. 2f). For each stimulation site we compared two sets of the responses evoked during first (1–15) and second (31–45) stimulation trials (Fig. 2a). Most of the overlaps was found in the range of 40–50 % indicating that most of responses evoked from single site are similar and does not show the selectivity in contrast to the responses from different sites with overlaps in the range of 0–25 % (see Fig. 2f red bars).

### Selectivity of the spike rate within small intervals

During the formation of single evoked bursts, the spike rate changed significantly, on the scale of tens of milliseconds. The maximum spike rate was observed at 50–100 ms after the stimulus artifact. In previous studies of spontaneous bursting activity, it has been shown that spiking patterns within a burst are organized in a non-random, repeatable manner (Madhavan et al. 2007; Raichman and Ben-Jacob 2008; Rolston et al. 2007). In particular, the spiking patterns at the beginning and end of the bursts displayed maximum reproducibility. Therefore, the analysis of selectivity with respect to the spiking activity over the entire response (total spike count) may not account for the unique activity features in different latencies of the burst (i.e., the beginning and end of the burst with high reproducibility and the middle with high variability). To overcome this, we analyzed the selectivity of the spike rate within small intervals (20 ms) by performing the following procedure.

**Fig. 3:**
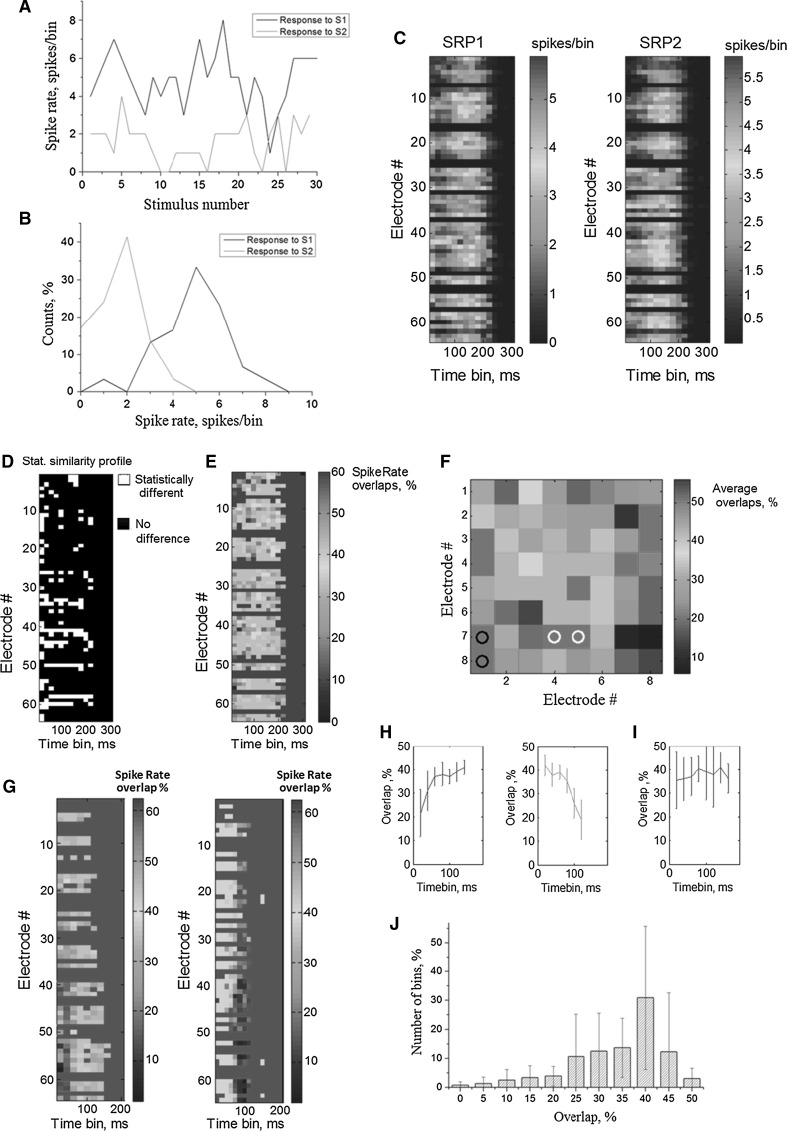
**a** Time courses and **b** distributions of spikes within single 20 ms time bins for a single electrode in response to the stimulation of sites S1 (*black line*) and S2 (*dashed red line*). **c** Spike rate profiles representing the average number of spikes registered in 64 electrodes for each 20 ms time bin after the stimulus. **d** *Statistical selectivity signature* of each time bin and electrode for the responses to two stimulation sites; the time bins of white color correspond to the statistically selective spike rate intervals. **e** Estimation of the overlap signature. The *color* grade corresponds to the overlap values (see “Materials and methods” section). **f** The average overlaps for the first 20 ms post-stimulus interval of the responses from each electrode according to its location on the MEA for one experiment. **g** The *overlap* s*ignature* for different cultures and stimulation sites. Highest selectivity (lowest overlaps) was observed in the beginning of the responses (*left image*) or at the end (*right image*). **h** Average overlaps for different time bins estimated for two cultures in **h** (*vertical lines*—standard deviation). **i** Average overlaps for different time bins estimated for all experiments (11 cultures, 50 trials). **j** The distribution of the overlap values within all post-stimulus time bins (11 cultures, 50 trials). (Color figure online)

Each 300 ms of the post-stimulus response for each electrode was divided into 20 ms bins. The responses from stimulation sites S1 and S2 for each electrode were characterized by the spike rate pattern (see “Materials and methods” section). Each pattern was presented as 2d matrix (64 electrodes × 15 time bins) of spike rate values. An example of the responses after each stimulus within a single bin for a single electrode is shown in Fig. 3a. The black and red curves depict the responses corresponding to stimulation sites S1 and S2, respectively, whereas their distributions are presented in Fig. 3b. Note that the responses in time bins where no spikes were elicited (zero values) were also taken into account because the entire response within 300 ms of the stimulus contained spikes and was classified as an active electrode. The average spike rate profiles for all electrodes are illustrated in Fig. 3c as color-coded images (left and right profiles representing the average spike rate patterns (SRP1 and SRP2) from the stimulation of the two sites, respectively). The color grade encoded the values of the average spike rate within each time bin and electrode.

Next, we compared the spike rates of the responses to different stimulation sites. All active electrodes and time bins were tested for significant differences between the two sets of responses (see “Materials and methods” section). The time bins with significant differences (*p* < 0.05) for the responses were defined as being *statistically selective*. A color-coded representation of the statistical selectivity for the time bins within a single experiment is shown in Fig. 3d (white color: statistically selective time bins; black color: no difference). A set of such binary indicators for all bins and electrodes can be treated as a *statistical selectivity signature,* emphasizing its uniqueness for a particular culture and specific stimulation sources.

Next, we analyzed the accuracy of these classifications. The spike rate overlap for the example shown in Fig. 3b was 10 %. A set of overlaps within each time bin for each electrode was defined as an *overlap signature* and is represented as a color pattern (Fig. 3e). The color of each time bin encodes the overlap value for that bin. In this example, the lowest overlap values were found in the initial time bins (20–40 ms after the stimulus artifact). Note that only a few electrodes had a relatively small amount of low (<20 %) overlaps. However, other cultures may show more efficient selectivity, i.e., have a higher number of low overlap time bins (Fig. 3g). In general, the spike rate measure with respect to small time bins (20 ms) revealed more variable results compared with the rates calculated for the entire evoked burst (300 ms). The number of electrodes having at least one bin with an overlap of less than 5 % was 64.2 ± 23.7 % of the total number of active electrodes, whereas the number of electrodes having a total spike count overlap of less than 5 % was 1.5 ± 5.4 % (11 cultures, 50 trials).

Furthermore, we considered whether the spatial organization of highly selective electrodes during a time bin had a non-random structure. We plotted the overlap values of the first time bin from each electrode according to its location on the MEA (Fig. 3f). Note that relatively low overlap values were localized in space non-randomly. This may indicate that a particular subnetwork or a cluster of neurons with high selective properties was activated in the culture. Surprisingly, the latencies (i.e., time intervals) of the overlap signatures relative to the cluster of bins with minimum overlaps (≤10 %) were found to be different for various cultures and stimulation sites (Fig. 3g). Interestingly, a high fraction of time bins with low overlap (high selectivity) could only be found at the beginning (Fig. 3h, left) or at the end (Fig. 3h, right) of the signature, i.e., burst intervals with high repeatability (Raichman and Ben-Jacob 2008; Pimashkin et al. 2011). However, on average, all cultures demonstrated a relatively stable selectivity during the response (Fig. 3i) which indicated that such intervals of high selectivity were unique to specific morphological or synaptic neural network grown in the culture. The distribution of the overlaps for all bins is shown in Fig. 3j (11 cultures, 50 trials). The fraction of time bins with an overlap of less than 10 % was 2.53 ± 3.67 % of all the bins that responded to both stimulation sites. The fraction of time bins with statistical selectivity was 35.34 ± 22.15 %. Each selective time bin (i.e., with low overlap value) could be considered as an indicator of a unique stimulation site.

### Selectivity of spiking patterns

In this section, we investigated the capability of a population of neurons to generate statistically separable responses to the stimulation of different sites. Several classification methods were applied to estimate the selectivity.

**Fig. 4:**
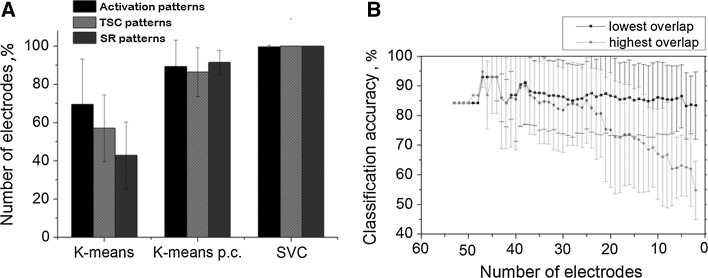
**a** Classification accuracy of population responses with respect to the stimulus response activation time, total spike count (TSC) and the first time bin spike rate (SR). The accuracy was estimated using K-means clustering, K-means clustering with predefined centroids and Support Vector Clustering methods (see “Materials and methods” section) (n = 11, 50 trials). *Error bars* represent standard deviation. For each characteristic the methods produced significantly different results (*t* test, *p* < 0.05) except K-means and K-means p.c. for activation patterns (*p* = 0.13). **b** Classification accuracy of the spike rate in the responses estimated by K-means clustering with predefined centroids using patterns comprised of different numbers of electrodes. *Black curve* patterns comprised of the electrodes with the highest overlap; *red curve* patterns comprised of the electrodes with the lowest overlap. (Color figure online)

First, we applied a standard K-means clustering method to estimate the classification accuracy (see “Materials and methods” section). The average classification accuracy for three different response characteristics is shown in Fig. 4a. The estimated accuracy for the spike rate in the first time bin was found to be insufficient for selectivity (42.92 ± 17.42 %). Note that for the burst activation time the accuracy was higher but not significantly different from the spike rate accuracy (69.46 ± 23.79 %).

Next, we applied a K-means clustering with predefined centroids (K-means p.c., see “Materials and methods” section). In this case, the efficiency of classification increased significantly compare to K-means (Fig. 4a). The average classification accuracy was 89.34 ± 13.81 % for burst activation, 86.47 ± 12.83 % for the total spike count (TSC) and 91.41 ± 6.39 % for the first time bin spike rate. Thus, the spike rates in the first time bin could be considered the most efficient for the selectivity but the differences for three characteristics were not statistically significant (ANOVA analysis).

Finally, we tested the classification accuracy using a support vector clustering **(**SVC**)** method (see “Materials and methods” section). The results demonstrated that the SVC in most cases revealed error free classification (Fig. 4a). The classification accuracy was 99.68 ± 0.74 % for the burst initiation profile, 100 % for the total spike rate and 100 % for the first time bin spike rate. Note that the result for selectivity with respect to the burst initiation profile is consistent with previous work by Shahaf et al. (2008). Our results clearly demonstrate that patterns composed of activity from all electrodes can be much more effective for selectivity than activity recorded from single electrodes. In summary all methods produced significantly different results considering each tested characteristic (*t* test, *p* < 0.05) except K-means and K-means p.c. didn’t show significant difference in comparing activation time patterns. The best results were found using SVC method for each tested response classification which were close to 100 %. SVC clustering basically estimates nonlinear boundaries of the clusters in characteristics space. Best clustering accuracy can be explained by the fact that consequent stimulation of single site may evoke several unique types of the response patterns. Multiple patterns (motifs) in the response sequence forms complex distribution of the response characteristics (spike rates, activation times) that cannot be separated by plane with linear classifiers (K-means and K-mean p.c.).

We also studied how the selectivity depended on the number of electrodes taken into the analysis and how sensitive it was relative to electrodes with higher spike rate selectivity. The classification accuracy of the total spike count measure was estimated by *k*-*means p.c.* clustering for all electrodes that responded to both stimulation sites. Then, the electrode with the lowest overlap (i.e., the highest selectivity) was excluded from the analysis and the accuracy was recalculated. This procedure was repeated until only two electrodes with the lowest *overlap* remained (Fig. 4b, black line). The result showed that the selectivity was independent of the number of electrodes with high overlaps. In other words, we found that the classification accuracy for two electrodes with the lowest overlaps was not significantly different to the maximum accuracy estimated for all electrodes. The same characteristic was calculated when electrodes with the highest overlaps (i.e., the lowest selectivity) were excluded iteratively (Fig. 4b, red line). One can notice that the difference between the two curves is visibly notable until 20-23 electrodes remained in the patterns. A significant difference between the two curves could be observed only in the range of 2–11 electrodes. In other words, the stimulus response activity recorded from a group of any 12 or more electrodes (up to 64) may be sufficient to decode the stimulus location with the highest accuracy.

## Discussion

We have demonstrated that cultured neural networks are capable of distinguishing input stimuli applied to different electrodes in a MEA system. We used mature hippocampal cultures of 20–30 days in vitro with a relatively stable functional organization of synaptic connections. The network responded selectively to different stimulus locations. In other words, spike trains that propagate in a culture network contain information about the stimulus location. Such a feature, referred to as selectivity, is one of the key functional properties of brain circuits used to classify sensory information (Birznieks et al. 2001; Cariani 2001; Heil 1997; Johansson and Birznieks 2004; Shahaf et al. 2008).

A necessary condition for the selectivity estimation in the cultures was a stability of the stimulus response characteristics (total spike count) during the experiment (Fig. 2a, b). Switch of the stimulation source didn’t affect on the train of the responses evoked from various sites. The mechanism of such stability could be explained by the fact that we used a low-frequency stimulation that was close to the natural frequency of spontaneous bursts, thus not affecting of the culture activity. In a previous work, response stability has been analyzed during long low-frequency stimulation of a single electrode under the same experimental conditions (Pimashkin et al. 2013). No significant changes were reported, even after 4–5 h of stimulation. Similar experiments using an open-loop low-frequency stimulation were done by Le Feber et al. (2010) and Marom and Shahaf (2002). In other studies, the stimulation of multiple sites did not significantly change the spatio-temporal characteristics of the responses (Chiappalone et al. 2008; Maeda et al. 1995). Only high-frequency stimulation significantly changed the first spike times and spike rate of the low-frequency responses (Tateno and Jimbo 1999; Chiappalone et al. 2008). It was also shown that a functional structure of connectivity in the culture was affected by consequent stimulation of two electrodes (Le Feber et al. 2015). Low-frequency electrical stimulation at one electrode disturbs the balance between activity and connectivity which induce new spiking pattern in the stimulus response. Such “adaptation” to the stimulus might occur in our experiments during testing of the stimulation sites before main stimulation trial (see “Materials and methods” section). Therefore we suggest that the selectivity experiments were performed in the cultures with stable functional connectivity.

We discovered that two principal characteristics of neural signaling in the stimulus response, such as the average spike rate and evoked burst activation time, were sufficient for the stimulus location retrieval. In previous studies, it has also been shown that the spike rate (Tessadori et al. 2012) and activation time characteristics (Shahaf et al. 2008) could be used for the estimation of selectivity in the stimulus response.

In addition to these two characteristics, population characteristics of multisite patterns, i.e., population coding, could be the most effective for location retrieval. In particular, the patterns of activation times and the order of the first spike occurrence from multiple electrodes provided 100 % accuracy in two stimulation site experiments (Shahaf et al. 2008). We also demonstrated that patterns comprised of spike rates could be used for high selectivity estimation (Fig. 4).

Basic cellular mechanisms of selectivity are associated with a certain synaptic organization of the network connectivity. On the one hand, the connectivity that spontaneously forms during the development of the culture allows for population burst discharges involving the activation of almost all neurons in the network. On the other hand, burst discharges are comprised of a number of synaptic signaling pathways that can be activated selectively by an appropriate stimulation. In particular, we demonstrated that the spike rate at certain time intervals (∆*T* = 20 ms) within the burst response could display significantly higher selectivity than for the entire stimulus response (300 ms). Analysis of the entire intra-burst structure showed that responses in different cultures have short phases or intervals in which selectivity was found in certain cultures. Despite the high repeatability of the spiking patterns at the beginning and the end of the evoked bursts (Pimashkin et al. 2011), we haven’t found high selectivity at these phases in all cultures in average which may be explained by high variability of the internal synaptic organization in different cultures. Also such selectivity features in different phases of the responses may depend on stimulus location in the network. In other words, there might be network sites preferable for better retrieval of input information. Such effects should will be explored in further studies.

For the comparison of two subsets of the responses from individual electrodes, we used statistical two-sample *t* test and K-means clustering with a cross-validation of the two samples. Such an approach can be used only for the selectivity estimation in experiments with two stimulation sources. However, K-means clustering can be used for this analysis as well as for the estimation of selectivity to many stimulation sites. To evaluate clustering results we used overlap measure which represents the percentage of the patterns that cannot be clearly associated with certain stimulation site. Such indicator can measure relative quality of the selectivity. The clusterization result can be also evaluated using the Davies–Bouldin index, which indicates how well clusters are separated. The dispersion of a cluster and dissimilarity between clusters are often used to compute the Davies–Bouldin index (Davies and Bouldin 1979). However we didn’t use it because such measure doesn’t represent actual percentage of the data that cannot be clearly identified with a certain cluster and can be useful in clustering estimation of multiple stimulation sites responses. The Support Vector Clustering method can also be used with much higher accuracy in the clustering due to the non-linearity of the algorithm, in contrast to the k-means method.

Finally, our method of stimulus characteristics decoding could be interesting for the design of bidirectional neural interfaces in vitro, in which reliable selectivity is one of the key problems (Carmena et al. 2003; Cozzi et al. 2005; Demarse et al. 2001; Doud et al. 2011; Lebedev et al. 2005; Luo and Sullivan 2010; Novellino et al. 2007; Shahaf et al. 2008; Warwick et al. 2010). The advantage of our approach is that we can identify specific electrodes and parts within the bursts that are highly selective for stimulation at different network sites. To estimate the selectivity, we applied a K-means clustering method that, in general, performs a linear classification. Such simple classification methods can be implemented for a closed-loop system with low response latencies and thus can be efficiently used for bidirectional interfaces.

## Electronic supplementary material

Below is the link to the electronic supplementary material.

Supplementary material 1 (DOCX 147 kb)
