# Supplementary material for: Selectivity of stimulus induced responses in cultured hippocampal networks on microelectrode arrays
Source: Cogn Neurodyn. 2016 Feb 22;10(4):287–99. doi: 10.1007/s11571-016-9380-6 (PMC4947052; doi:10.1007/s11571-016-9380-6)
Supplement: Supplementary file 1 — Supplementary material 1 (DOCX 147 kb) [file 11571_2016_9380_MOESM1_ESM.docx]

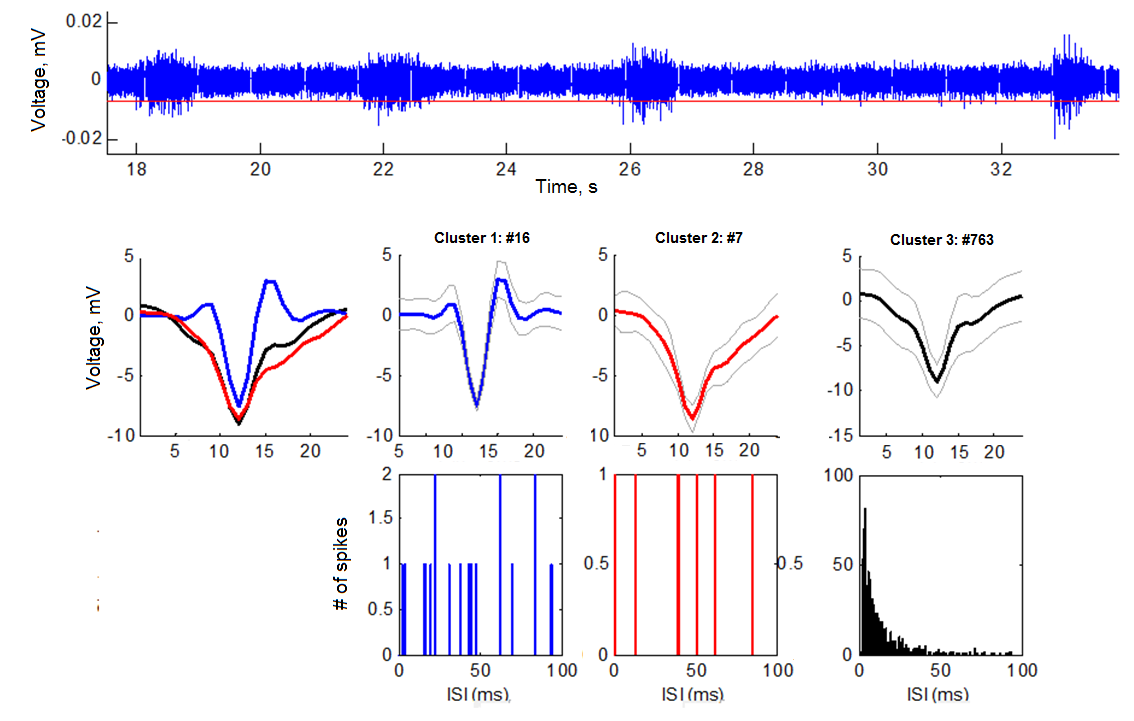


**Support figure 1**. Spike shape analysis of signals recorded from single electrode. Spikes between the bursts had unique shapes (cluster #1 and #2). Most of the spikes contributed to the bursts had similar shape (cluster #3).
